# The complete mitochondrial genome of *Conus striatus* (Neogastropoda: Conidae)

**DOI:** 10.1080/23802359.2016.1192502

**Published:** 2016-07-11

**Authors:** Po-Wei Chen, Sheng-Tai Hsiao, Kao-Sung Chen, Chen-Te Tseng, Wen-Lung Wu, Deng-Fwu Hwang

**Affiliations:** a Department of Food Science, National Taiwan Ocean University, Keelung, Taiwan;; b Marine Fisheries Division, Fisheries Research Institute, Keelung, Taiwan;; c Planning and Information Division, Fisheries Research Institute, Keelung, Taiwan;; d Biodiversity Research Center, Academia Sinica, Taipei, Taiwan

**Keywords:** *Conus striatus*, mitogenome, next-generation sequencing

## Abstract

*Conus striatus* is a kind of piscivorous cone snail. We have sequenced it by next generation sequencing method. We used *de novo* assembly and reference mapping methods to assemble mitogenome. The mitochondrial genome is 15,738 bp, containing 13 protein coding genes, 22 transfer RNAs and 2 ribosomal RNAs genes. The overall base composition of *C. striatus* is 25.9% for A, 16.3% for C, 20.8% for G and 38.6% for T. The phylogenetic analysis was conducted with 18 related species and confirmed the classification status. The complete mitogenome of the *C. striatus* provides an essential and important DNA molecular data for further phylogeography and evolutionary analysis for cone snail phylogeny.

*Conus* is a kind of sea snails which are predators and venomous animals. They use their harpoon-like radular teeth to inject the venom to anesthetize the prey. There are about 600 different cone snails that are classified according to their feeding behavior into piscivorous, molluscivorous and vermivorous species (Le Gall et al. 1999). The cone snail *C. striatus* (Linnaeus 1758) belongs to a clade of piscivorous snails from the Indo–Pacific. They have been observed to elicit a spastic paralysis upon injection of venom into a fish during prey capture (Kelley et al. 2006).

Specimens of *C. striatus* (voucher no. 20150207-002) were collected from Penghu, Taiwan (23.564N, 119.693E) and deposited in Marine Toxins Lab., Department of Food Science, National Taiwan Ocean University. The total genomic DNA was extracted from muscle using magnetic bead technique with the KingFisher magnetic processors (ThermoFisher Scientific Inc., Worcester, MA). The raw next-generation sequencing reads generated from MiSeq sequencer (Illumina, San Diego, CA) were *de novo* assembled and reference mapping was conducted by commercial software (Geneious V9, Auckland, New Zealand) to produce a single circular form of complete mitogenome with about an average 40.8 coverage (2244 out of 6,214,252 reads, 0.036%). The complete mitochondrial genome of *C. striatus* is 15,738 bp in size (GenBank: KX156937), including 13 protein coding genes, 22 transfer RNA genes and 2 ribosomal RNA genes. The overall base composition of *C. striatus* is 25.9% for A, 16.3% for C, 20.8% for G and 38.6% for T. The protein coding rRNA and tRNA genes of *C. striatus* mitogenome were predicted using MITOS (Bernt et al. 2013) and tRNAscan-SE (Schattner et al. 2005).

We used MEGA 6 (Tamura et al. 2013) to construct the phylogenetic relationships of the *C. striatus* and related families by Neighbour-joining method with 1000 bootstrap replicates based on the 13 protein-coding genes and 2 ribosomal RNA genes of the other 20 complete mitochondrial genomes of Neogastropoda marine mollusks, which are reported in Genbank of NCBI database. Bootstrap support values were relatively high, with 13 nodes having values >95% and 8 nodes demonstrating 100% bootstrap support (Figure 1). *C. striatus* was grouped together with six other *conus* species from the family Conidae. The lineages of Conidea are strongly supported in this report and are agreed with previous studies (Bouchet et al. 2011; Puillandre et al. 2014).

**Figure 1.**
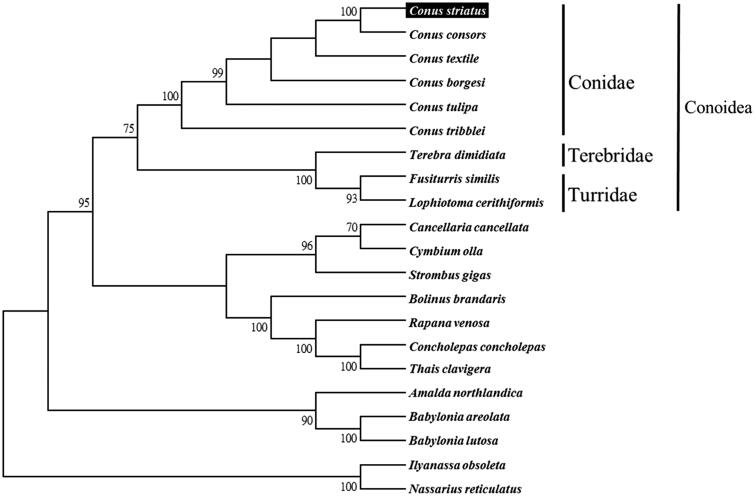
Phylogenetic tree generated using the Neighbour-joining method with 1000 bootstrap replicates based on complete mitochondrial genomes. *C. striatus* (KX156937), *C. consors* (KF887950), *C. textile* (DQ862058), *C. borgesi* (EU827198), *C. tulipa* (KR006970), *C. tribblei* (KT199301), *Terebra dimidiate* (EU827196), *Fusiturris similis* (EU827197), *Lophiotoma cerithiformis* (DQ284754), *Cancellaria cancellata* (EU827195), *Cymbium olla* (EU827199), *Strombus gigas* (KM245630), *Bolinus brandaris* (EU827194), *Rapana venosa* (KM213962), *Concholepas concholepas* (JQ446041), *Thais clavigera* (DQ159954), *Amalda northlandica* (GU196685), *Babylonia areolata* (HQ416443), *B. lutosa* (KF897830), *Ilyanassa obsoleta* (DQ238598) and *Nassarius reticulatus* (EU827201).
